# Vaccination—Letter from Dr. J. M. Toner

**Published:** 1873-02

**Authors:** J. M. Toner

**Affiliations:** Washington, D.C.


VACCINATION—LETTER FROM DR. J. M. TONER.—We have the pleasure of laying before our readers part of an interesting letter from Dr. J. M. Toner, of Washington City, upon vaccination. We know the thoughts and suggestions of Dr. Toner will be appreciated by them:

WASHINGTON, D.C., January 21, 1873.

*W. T. Goldsmith, M.D.:*

DEAR SIR,— . . . You and every well-informed physician are aware how important a factor, in securing the full protection afforded by vaccination, is the regularity of the different stages of the vaccine disease. An accident or violent disturbance to the point of insertion may convert what, under favorable circumstances, would be regular and protective, into an unprotective or spurious vaccination.

A good protective vaccination is not the simple unscientific operation that the unskilled public have come to consider it. Vaccination properly performed with fresh, active virus, and that process undisturbed through its stages of development, maturation and decline, unquestionably gives a person all the protection against small pox that Jenner ever claimed for it—which was, that it gives the same protection that inoculation or that small pox itself gives.

Although I inclose you part of a crust of non-humanized vaccina directly from the cow, sent me by Dr. Ephriam Cutter, of Woburn, Massachusetts, I hold, as he does, and the profession generally, that the evidence is wanting to prove that vaccination, by its transmission through human beings, has lost any of its protective power. This phase of the question has not been precipitated upon the attention of the profession, for it has engaged the thoughts and been the subject of experiment almost from the period of the discovery of the protective power of vaccination.

But there are a class of accidents that may, and often do, occur at different stages, both in the operation of inserting the virus and during the course of its development, performed with good matter and by a competent person. For instance, if by accident a puncture, cut or laceration is made so that blood flow to form in drops instead of a mere abrasion which permits merely the liquor sanguinis to exude and opens the absorbents, it will wash the virus out and away from the absorbents, where it will be mixed with the blood, when it will dry, crumble off and be lost. Should it be a secondary vaccination, there is danger that a hasty deduction might be made that the case resists the influence of the vaccine virus, and is, therefore, protected by his first vaccination against small pox. The human system will only be effected by vaccine virus in a soft or moist state, and in ordinary vaccinations this occurs in a very brief period after it is introduced. Another class of accidents which I apprehend occurs more frequently than is generally supposed is, after active virus is properly and successfully introduced, the regular course of the disease is interrupted after the third day, or from the time the first redness begins to appear, by the friction of badly-fitting clothes by rubbing, bruising, scratching, etc., that may break the distinctive vesicle that must form in every protective vaccination.

Danger from this class of accidents is imminent until after the twelfth day, when the vesicle has filled properly with pearly white lymph, has shrunken, and the drying stage has set in.

The abstraction of the pellucid lymph before the ninth day, or before the areola has formed—which denotes absorption—endangers, if it does not wholly prevent protection, no matter how marked may be the other evidences of constitutional disturbances.

When this accident occurs, I consider it to be the duty of the physician to revaccinate without delay. The only mode in this as in every case, for the physician to determine whether a given case is protective, is by the revaccination of active vaccine virus until the system, after repeated trial, will cease to respond to its presence. Yours truly, J. M. Toner.

